# Averaging sleep spindle occurrence in dogs predicts learning performance better than single measures

**DOI:** 10.1038/s41598-020-80417-8

**Published:** 2020-12-31

**Authors:** Ivaylo Borislavov Iotchev, Vivien Reicher, Enikő Kovács, Tímea Kovács, Anna Kis, Márta Gácsi, Enikő Kubinyi

**Affiliations:** 1 Department of Ethology, ELTE Eötvös Loránd University, 1117 Budapest, Hungary; 2 Institute of Cognitive Neuroscience and Psychology, Research Centre for Natural Sciences, 1117 Budapest, Hungary; 3 MTA-ELTE Comparative Ethology Research Group, 1117 Budapest, Hungary

**Keywords:** Circadian rhythms and sleep, Learning and memory

## Abstract

Although a positive link between sleep spindle occurrence and measures of post-sleep recall (learning success) is often reported for humans and replicated across species, the test–retest reliability of the effect is sometimes questioned. The largest to date study could not confirm the association, however methods for automatic spindle detection diverge in their estimates and vary between studies. Here we report that in dogs using the same detection method across different learning tasks is associated with observing a positive association between sleep spindle density (spindles/minute) and learning success. Our results suggest that reducing measurement error by averaging across measurements of density and learning can increase the visibility of this effect, implying that trait density (estimated through averaged occurrence) is a more reliable predictor of cognitive performance than estimates based on single measures.

## Introduction

Sleep spindles are thalamocortical transmissions^1,2^ observed mostly in mammalian non-REM sleep^3^ as brief (0.5–5 s^4^) trains of symmetric waves^5^ in the EEG signal. Different propositions for their defining frequency (waves/second) overlap in the 9–16 Hz band among humans^6,7^, mice^8^, and dogs^9–11^.

The most often reported cognitive correlate of sleep spindles in humans is a positive relationship with post sleep-recall (learning success). However, this has almost exclusively been reported in smaller samples^12–23^, and thus the reliability of this effect requires a stronger confirmation. On one hand, the study using the largest to date sample could not find such an association^24^. Moreover, it is troublesome that different studies use different algorithms^2^, since automatic spindle detection methods diverge in their estimates of spindle occurrence^25^. On the other hand, invasive work in animal models has revealed putative mechanisms^26^ to explain how spindles promote memory consolidation, as well as implicated causality^27^ where human data mostly allows only for correlation. The issue thus remains controversial.

Here we report a replication analysis for the link between spindle occurrence and learning in dogs. The dog (*Canis familiaris*) is a fairly new model species in sleep spindle research, but one advantage in addressing the problem of replicability is that currently only one method for detecting canine spindles has been consolidated across all published studies (from one single research group, Iotchev et al.^9–11^). The same method, adopted from the human literature^28^ with minor alterations for use in dogs^9^, will also be used here to avoid the problem of automatic detector divergence^25^. Moreover, the current analysis will include all unpublished data sets available to us which are fit for this analysis. This is crucial because in the human literature publication bias is suspected to underlie effects reported for (fast) spindle density^29^. The goal of the analyses presented here will be to evaluate if the relationship between sleep spindle occurrence and learning success is real or a type I error. To evaluate this, we will look both at the prevalence of positive findings and the conditions under which a positive or negative finding is observed. We will thereby also compare the prevalence of associations between (1) single and (2) averaged measurements of sleep spindle density (spindles/minute) and learning, the latter being deemed more likely to reflect underlying traits and to be freer of measurement error.

## Methods

### Procedure

Three datasets were included in the analyses. In all datasets the basic learning paradigm (“novel words paradigm”) was based on the one used to obtain the data for Iotchev et al.^9^ and Kis et al.^30^ (for details see Supplementary Material). We included the dataset used in these studies for comparison (data set 0, N = 15). Data set 1 (N = 19) originated from Reicher et al., in prep and data set 2 (N = 13) from Kovács et al. in prep. One dog participated in data set 0 and 2, otherwise the samples did not overlap. Before sleep, dogs were required to learn novel words (in English), and associate them with actions that they had been trained to perform to different verbal commands before (in Hungarian). After sleep, the final performance was measured as the percent of correct trials (out of eighteen, on the re-test), and learning gain (% performance re-test minus test) was also calculated. All three datasets were comprised of an adaptation sleep, followed by two counter-balanced, repeated-measures conditions. Condition 1a used a supportive type of training (using both food and social reward in case of correct action and no scolding in case of incorrect action), while in condition 1b a controlling type of training was conducted by a different experimenter (using only food reward without social reinforcement in the case of correct action and scolding in case of incorrect action). In condition 2a training was carried out by the owner in a socially relevant manner (using both food reinforcement and social reward in case of correct action), in condition 2b training was carried out by an experimenter unknown to the dog in a socially irrelevant manner (using food reinforcement but without social reward in case of correct action).

### Subjects

A total of 46 dogs (23 females, age range 1–9 years, 28 were purebred representatives from 16 different breeds) participated in the three studies (0, 1, 2). One dog was included in both study 0 and 2 (a female golden retriever, aged 1 year in study 0 and 2 years in study 2). Because search for sleep spindles was restricted to non-REM sleep as in Iotchev et al.^9,10^, dogs which did not reach this stage or had otherwise corrupted or missing files were assigned missing values for sleep spindle density. During adaptation (occasion 1), three dogs were assigned missing values in study 0, five dogs in study 1, and two dogs in study 2. Regarding data from experimental conditions (occasion 2 and 3), two missing values were assigned in condition 1a, and one in condition 2a (see conditions below). Missing values were excluded from our analyses and the calculation of averages.

### Ethical statement

According to the Hungarian regulations of animal experimentation, our non-invasive polysomnography research does not qualify as an animal experiment. The Hungarian Scientific Ethical Committee of Animal Experiments issued a permission (under the number PE/EA/853-2/2016) approving of our non-invasive protocol. All owners volunteered to participate in the study and were informed about the procedure before beginning.

### Electrode placement and EEG post-processing

**Figure 1:**
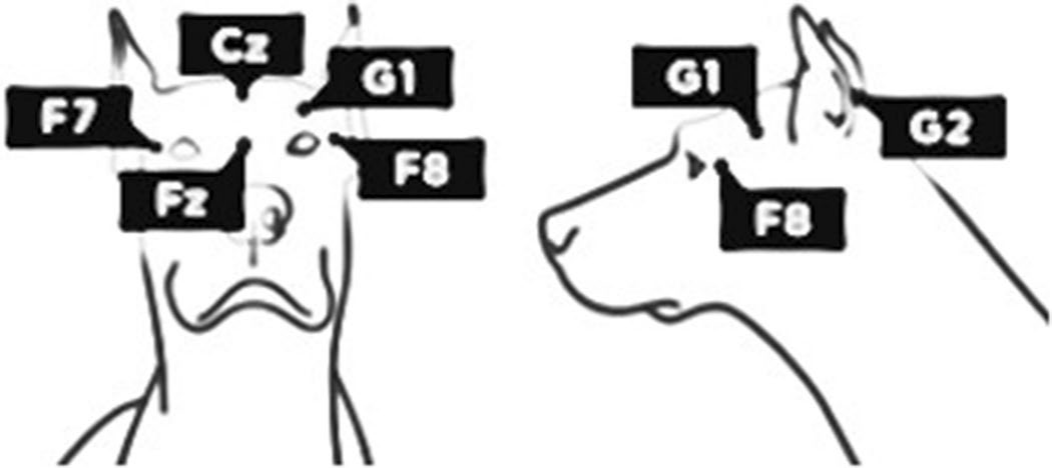
Schematic drawing (by Vivien Reicher) of electrode placement in the dog identical in study 1 & 2 (study 0 used the same electrode placement, but without the F7 channel).

Electrode placement (see Fig. 1) followed the method outlined by Kis et al.^31^ The polysomnographic recordings were manually categorized into sleep stages (wake, drowsiness, non-REM, REM, see Supplementary for example images) according to standard criteria^31^ (validated in Gergely et al.^32^). The traces identified as non-REM (descriptive statistics in the Supplementary) were scanned for spindles using the frontal (Fz) and central (Cz) midline electrodes.

### Spindle detection

Automatic detection was implemented as in Iotchev et al.^9^ on parts of the signal marked as non-REM sleep and pre-filtered between 5 and 16 Hz. Specifics of the applied algorithm are detailed in Iotchev et al.^9,10^ and based on similar criteria validated against visual experts on human EEG by Nonclercq et al.^28^. Importantly, the algorithm invokes 2 steps, as initial detections are used to re-calculate boundaries for the target amplitude and frequency of spindles for each dog and recording, in line with the assumption that these are normally distributed within individuals. In the first step the frequency is assumed to be 9–16 Hz and a minimum amplitude criterion of more than 1 standard deviation above the average of the searched signal is set. For the individual adjustment in the second step the algorithm calculates maximum likelihood estimates for the means and standard deviations of amplitude and frequency. The amplitudes and frequency of the final detections have to be within 2 standard deviations of the estimated means.

### Analysis

Since only Fz was active in data set 0, we will focus below mainly on results obtained from Fz. We will also refer to and discuss detections from across the whole 9–16 Hz frequency range, considering that many studies looking into spindle–learning correlations did not divide spindles into slow and fast ones^12–14,33^. We refer to Supplementary Tables S1, S2, S4 and S5 for statistics obtained across both electrodes, and spindle sub-types (slow and fast). All associations were tested with Pearson correlations using SPSS v25.

## Results

**Figure 2:**
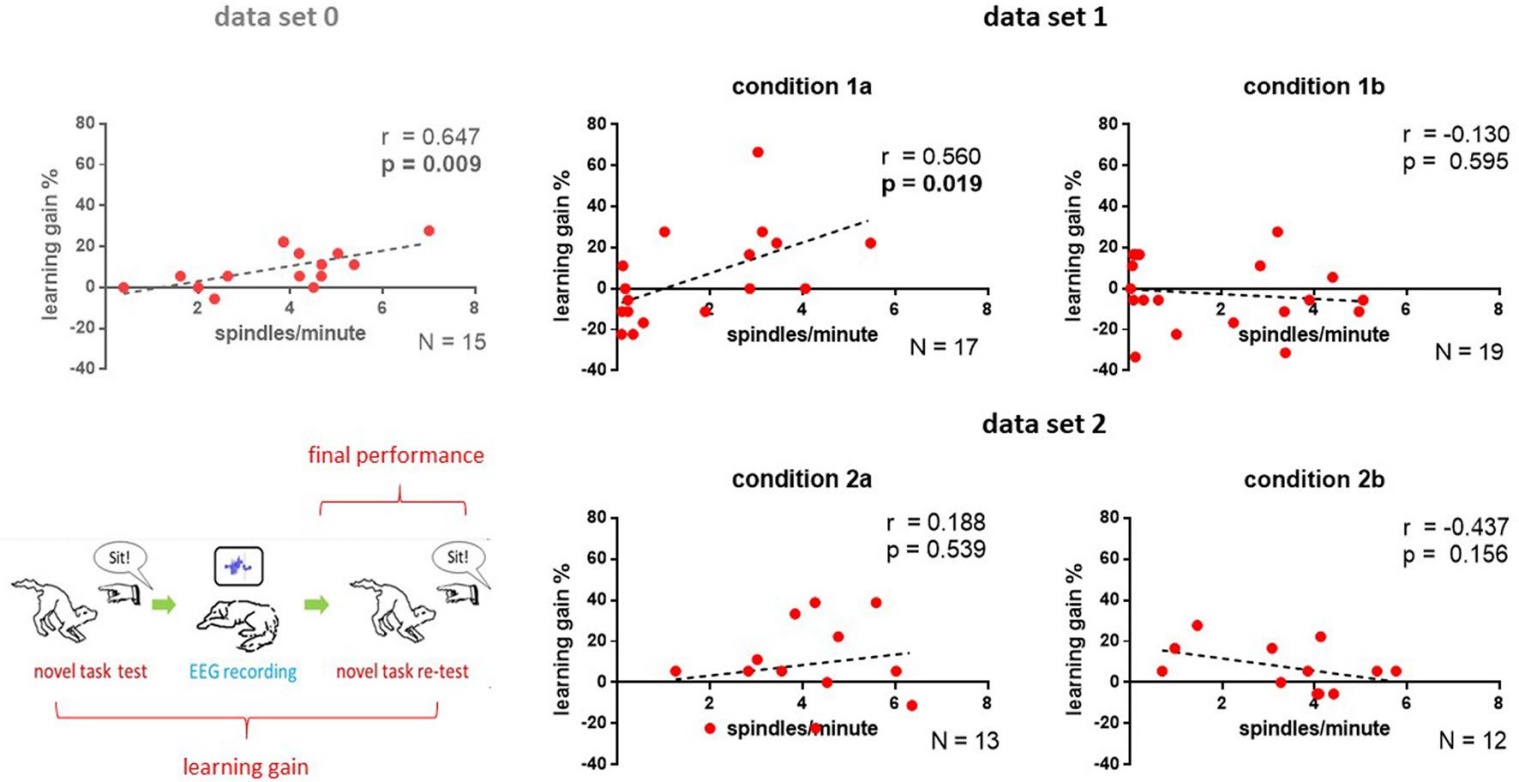
Associations between spindle density (spindles/minute) and learning gain as measured over the frontal midline electrode (Fz) in data sets 0, 1, and 2. For the already published finding in data set 0^9^ we used lighter colors. Two dogs did not sleep in condition 1a and one dog did not sleep in condition 2b, these animals were excluded from the analysis. Schematic drawing (by the corresponding author) of how learning gain was calculated during tests on the novel task.

By comparing single measurements (i.e. from one single attendance, same condition) of sleep spindle occurrence and learning success, we found an association between spindle density and learning gain for data sets 0 and condition 1a, but not for condition 1b and data set 2 (see Fig. 2). Upon closer examination, both effects were specific to the slow spindle type (Table S2) and for condition 1a a trend for the same effect was observed on Cz, as well (Table S1).

Next, we compared averaged measurements (across attendances/conditions) of sleep spindle occurrence and learning, to test how reducing measurement error could affect the relationship between spindle density and learning in the three data sets, considering that a single measurement for each variable might be less reliable^34^. Averaging across attendances/conditions was deemed valid, because one of the rationales for this analysis was to approximate the underlying traits, rather than estimate a condition-specific expression of either spindle occurrence or learning performance. Therefore, we averaged within each data set and for each dog spindle density values obtained from all three recordings (in data set 0 these correspond to the adaptation, control and learning conditions, while in data set 1 and 2 there was only an adaptation and two learning conditions; due to missing values, for some dogs the averages were based on only two recordings, see Supplementary for further details). We furthermore averaged learning performance variables (final performance and learning gain) for the two learning conditions in data sets 1 and 2 (this was not possible for data set 0 in which there was only one measure for each). We tested associations with both (averaged) learning gain and (averaged) final performance because their distribution and range (Table S3) suggested that for each data set a different read-out variable might better reflect the underlying learning process.

**Figure 3:**
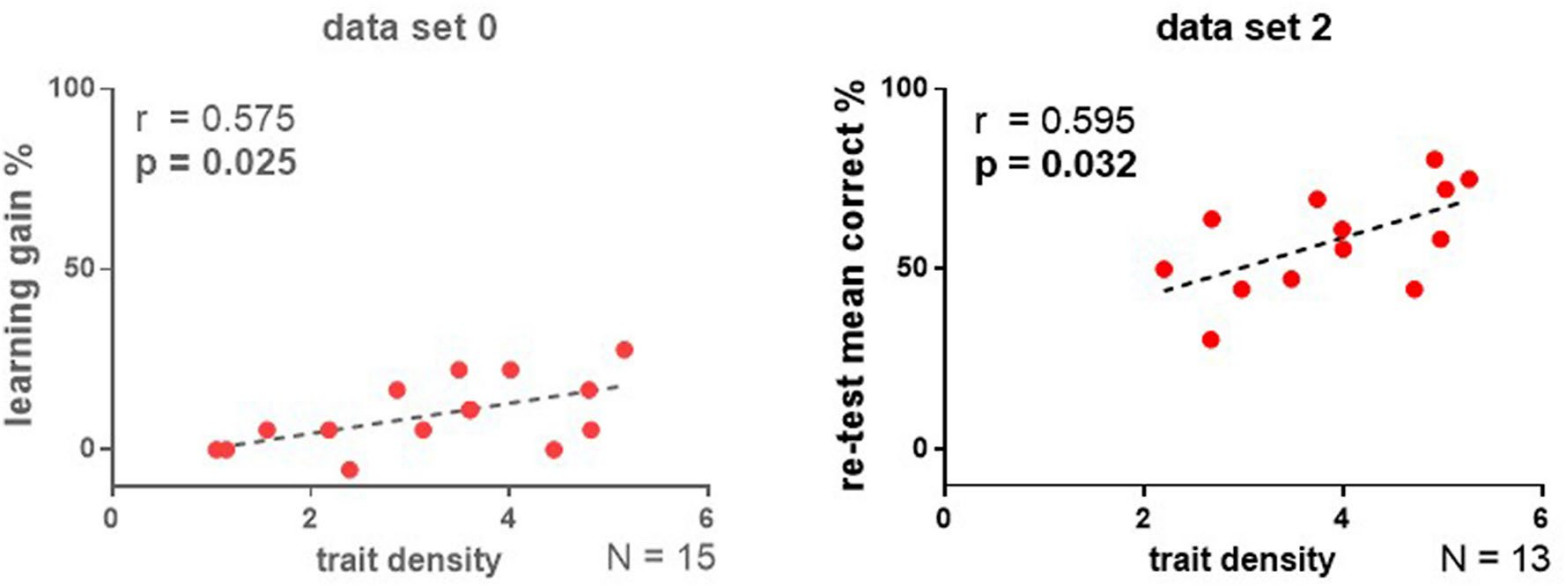
Associations between trait density (estimated here by averaging across recordings) and learning success (also averaged for data set 2, based on final performance) over the frontal midline electrode (Fz) in data sets 0^9^ and 2. Averaging learning gain was not possible in data set 0 as the experiment consisted of only one learning condition. Data from the published data set 0 is presented in lighter colors.

Averaged density was positively associated with learning gain in data set 0 and average final performance in data set 2 (Fig. 3). These effects were also significant for the slow sub-type and specific to Fz (Tables S4, S5). Neither averaged learning performance variables were associated with averaged density in data set 1, but note that in condition 1b, more than half of the dogs (57.9% or 11 out of 19 animals) worsened their performance on the novel task after sleep. In comparison, only 7 dogs (36.8%) did so in condition 1a. Together with a visual inspection of the data (Fig. 1) and the performance overview provided in Table S3 (lowest average values for learning gain and final performance), these numbers suggest a floor effect on learning success in condition 1b.

## Discussion

A positive association between dogs’ spindle occurrence and learning success could be demonstrated in each of the three data sets. As a note of caution, different transformations of the raw variables (like averaged scores) and operationalization of learning (learning gain versus final performance) were used between some of the comparisons, while all available samples are also small (N < 20). An argument for treating the positive associations as cumulative derives from using the same detection algorithm^9^, and the same experimental operationalization of learning (the “novel words paradigm” of Kis et al.^30^) across all experiments. Intriguingly, all significant associations were specific to slow and frontal spindles (see Supplementary Tables S2, S4, S5) which resembles what is seen in humans when learning is tested with verbal material, like word-pairs^13,16,23^. In further support of the spindle-learning association, Type II errors are common in small, and thereby likely underpowered samples^35^. False negatives are also likely, considering that memory consolidation is not restricted to sleep alone in neither humans nor dogs^30,36^. Also, many additional conditions are known to influence if any effect is observed, e.g. relative timing to ripples and slow-waves^37^, emotional arousal^38^ and exact stage of non-REM sleep^14^. However, since in most animals it is hard to separate non-REM sleep stages from each other^31,39^ not all of these conditions can be tested outside of humans.

Surprisingly, although sleep-dependent memory consolidation operates in the time-frame of a single day^40,41^ and exposure to new information has been shown to result in direct increases in spindle occurrence in humans^15,23^, rats^33^ and dogs^9^, our results for data sets 0 and 2 suggest that estimating trait density by averaging across recordings might increase the visibility of spindle-learning associations. Other arguments for the predictive utility of trait density come from reports of stable spindle occurrence across nights in humans^42,43^, the heritability of sleep spindle density^44^, and the observation that different psychiatric conditions and natural aging, each associated with memory problems, can measurably reduce spindle occurrence in humans^45–49^ and specifically the occurrence of slow spindles in dogs^10^.

We conclude that the here examined data-sets lend additional support to the positive association between sleep spindle occurrence and learning observed in dogs earlier^9^, but the need for further evidence is not exhausted. Even more and larger samples will be required to establish to what extend low power accounts for the proportion of null results. Moreover, since trait occurrence is also associated with general mental ability^50^ more studies with a control condition, in which sleep is not preceded by learning demand, will be needed in dogs to separate if these correlations reflect memory consolidation or general learning potential.

## Supplementary Information

Supplementary Information 1.

Supplementary Information 2.
