# Supplementary material for: Averaging sleep spindle occurrence in dogs predicts learning performance better than single measures
Source: Sci Rep. 2020 Dec 31;10:22461. doi: 10.1038/s41598-020-80417-8 (PMC7775433; doi:10.1038/s41598-020-80417-8)
Supplement: Supplementary file 1 — Supplementary Information 1. [file 41598_2020_80417_MOESM1_ESM.docx]

**Supplementary**

Averaging sleep spindle occurrence in dogs predicts learning performance better than single measures

Ivaylo Borislavov Iotchev^a^*^, Vivien Reicher^a^^, Enikő Kovács^ab^^, Tímea Kovács^a^, Anna Kis^b^#, Márta Gácsi^a^#, Enikő Kubinyi^a^#

^a^ Department of Ethology, Eötvös Loránd University ELTE, 1117 Budapest, Hungary

^b^ Institute of Cognitive Neuroscience and Psychology, Research Centre for Natural Sciences MTA, 1117 Budapest, Hungary

*corresponding author

^first author contributing equally to this work

#senior authors contributing equally to this work

**Behavioral paradigms**

**Shared structure of study 0-2.**

*General outline*

The dogs and owners were invited on three occasions. The second and third occasion consisted of (1) training, (2) test, (3) polysomnographic recording, (4) re-test.

On occasion 1 (adaptation), dogs were familiarized with the polysomnographic recording set-up, electrode placement and room. It was tested whether the animals could successfully perform the four tasks known by the dog, each in response to both only a hand signal and to only the verbal command (in Hungarian, which was the original language of training). Reaching a 75% success rate was a precondition for participating in further phases of the study. The tasks were chosen based on the criteria that they can be executed without an object, with the owner (/trainer/experimenter) standing straight in front of the dog, and were well-trained (high rate of correct execution according to owner reports). The adaptation concluded with a polysomnographic recording to eliminate the “first-night effect” described in humans^4^ and dogs^5^.

The 2^nd^ and 3^rd^ polysomnographic recordings were obtained during the experimental conditions, indicated as 1a and 1b for data set 1, and 2a and 2b for data set 2. In each study, the dog underwent both conditions (in a counterbalanced order), following a within-subject design and each condition was tested on a separate occasion/attendance.

1. *Training*

In each experimental condition, the dog was trained to execute two known tasks in response to novel commands (in English). The training session always followed the same pattern and contained at least 4 blocks with 4 breaks. To continue to the next block (following prescribed breaks), the dog had to execute 10 correct trials in the preceding block:

(1.1) Executing the first task; verbal command + hand signal (block 1)

(1.2) 5-minute-long break in an adjacent room (break 1)

(1.3) Executing the first task; only verbal command (block 2)

(1.4) 10-minute-long break in an adjacent room (break 2)

(1.5) Executing the second task; verbal command + hand signal (block 3)

(1.6) 5-minute-long break in an adjacent room (break 3)

(1.7) Executing the second task; only verbal command (block 4)

(1.8) 15-minute-long break, the owner took the dog for a short walk (break 4).

1. *Test*

The baseline test, consisted of 18 trials on the two newly learned English commands (9 trials each) following the 15 minutes long break (1.8).

1. *Polysomnographic recording (see Kis et al.*^6^*).*
2. *Re-test*

Dogs were retested applying the same procedure as in the baseline test (1.9). Final performance was calculated as the percentage correct trials and learning gain as the difference (re-test minus test, using again percentages).

**Specific structure of study 0-2.**

Study 0 (data set 0)

Polysomnographic recordings lasted for 3 hours. In study 1 and 2 the overall procedure was closely modeled on the one developed by Kis et al. (2017)^7^.

Study 1 (data set 1)

Polysomnographic recordings lasted for 2 hours.

Dog trainers undertook the training part, while the experimenter tested the dogs’ post-training performance before and after the polysomnographic recordings. Two trainers were used for each dog and switched (in counterbalanced order) between conditions. A cage filled with meat and toys (acting as a distractor to lure the dog) was placed in the laboratory during the training. The dog trainer could initiate the teaching if the dog sat and looked at them, being at least 1.5 meters away from the distractor. The distractor was introduced to increase the number of trials that would require feedback from the trainer by increasing the likelihood of errors. This was relevant since the feedback type was a defining feature of each condition (see below). No distractor was used in the study from which data set 2 was obtained, but the owner was one of the trainers (condition 2a), and also tested the dog’s performance before and after sleep.

Dogs were required to learn the English words for two of the four commands on each occasion/condition. The 4 commands were specifically one of the following: stand (a), lay down (b), turn around (c), give paw (d). Different command-task pairs were offered in a randomized order between conditions.

A step in which the combined verbal command and hand signal were used preceded the other two steps, resulting in a total of 12 trials per block,

*Condition 1a*

For correct trials: The trainer could use a combination of verbal and social reinforcement, depending on the dog’s preference. For each correct action a treat was provided and then the trainer went around the cage.

For error trials: The trainer moved away so that the dog followed her and was again in the starting position, then the same command was repeated (aiding with the hand signal if necessary) until the dog executed the task properly, then the trainer praised it and gave it a treat. If the dog did not perform the correct action (without hand signal) after 10 trials, an extra break was given to the dog. Following the break, if the dog did not perform the correct action after 10 trials, the experiment ended and the dog was requested to repeat the session on another occasion (occurred in the case of two dogs).

For non-compliance: If the dog went to the cage and did not leave it when the trainer called it kindly, the trainer tried to lure the dog with food and without using the command “Nem!” (= No!). If the dog could not be lured away after 4 minutes, the trainer covered the cage with a blanket (occurred in case of one dog).

*Condition 1b*

For correct trials: The trainer gave the dog a treat (no praise) and went around the cage.

For error trials: The trainer scolded the dog, then moved away, so that the dog followed her and was again in the starting position. Then the trainer repeated the same command (aiding with the hand signal if necessary) until the dog executed it correctly, and gave it a treat without praising. If the dog did not perform the correct action after 10 trials (without hand signal), the procedure was the same as above.

For non-compliance: If the dog went to the cage and did not leave when the trainer called it firmly, the trainer inhibited the dog with a strict command.

Study 2 (data set 2)

Polysomnographic recordings lasted for 3 hours.

Dogs were required to learn the English words for two of the four commands on each occasion/condition. The 4 commands had been chosen from a wider range based on the previously described criteria. Different command-task pairs were offered in a randomized order between conditions.

Tests with the known commands were incorporated in the training protocol as two blocks of each 4 trials (alternating structure: ABAB) preceding the actual training as a warm-up (thus resulting in a total of 6, instead of 4 blocks), moreover the previously described breaks between blocks were omitted. Only the verbal commands were used in the first of these blocks and verbal commands + hand signals in the second. Moreover, during the later post-sleep retest on the novel commands in this study, 18 trials with the known commands preceded the actual testing to assure the dogs were properly awake and alert for the retest. *Condition 2a*

Training (and testing) were executed by the owner of the dog. Moreover, feedback to correct trials consisted of both treats and praise.

*Condition 2b*

Training (and testing) were executed by an unfamiliar experimenter. Feedback to correct trials consisted only of treats.

**Example Polysomnography**

*Wakefulness*


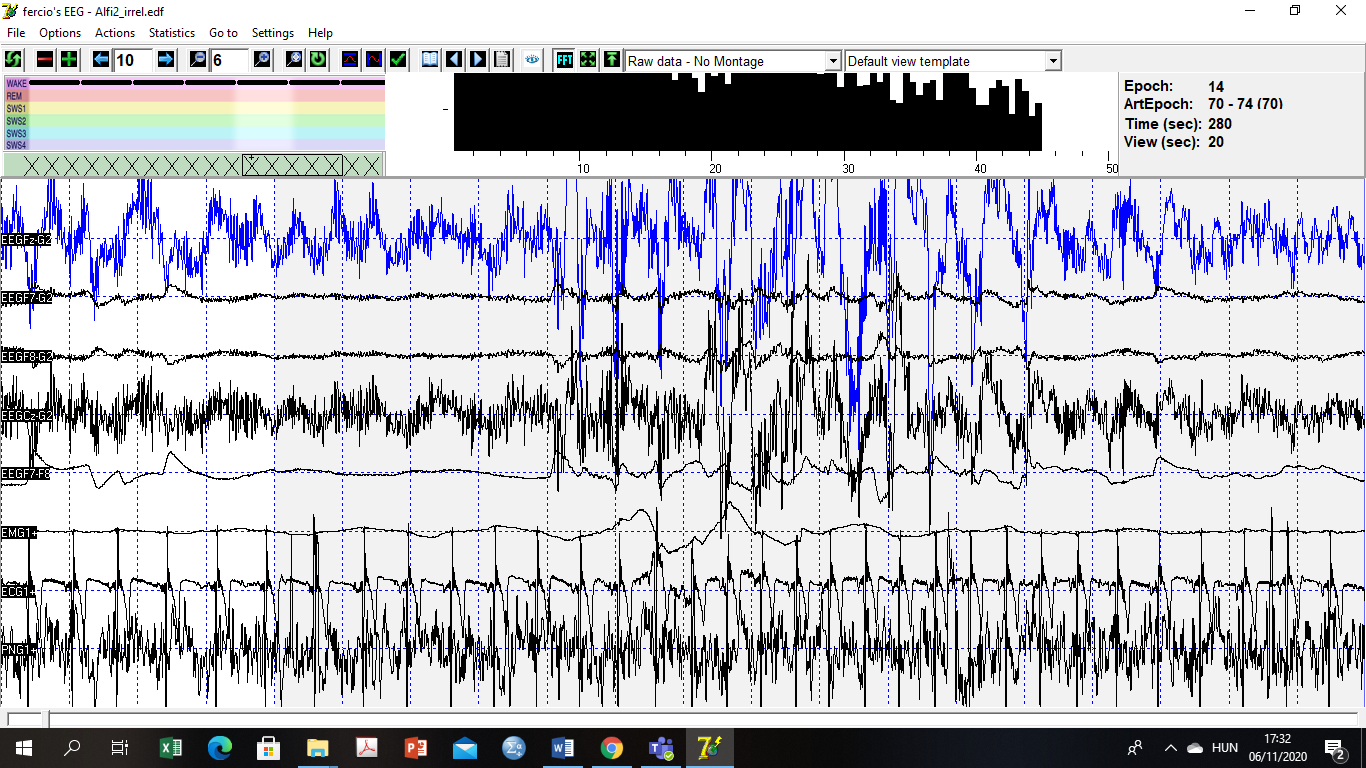


*Drowsiness*


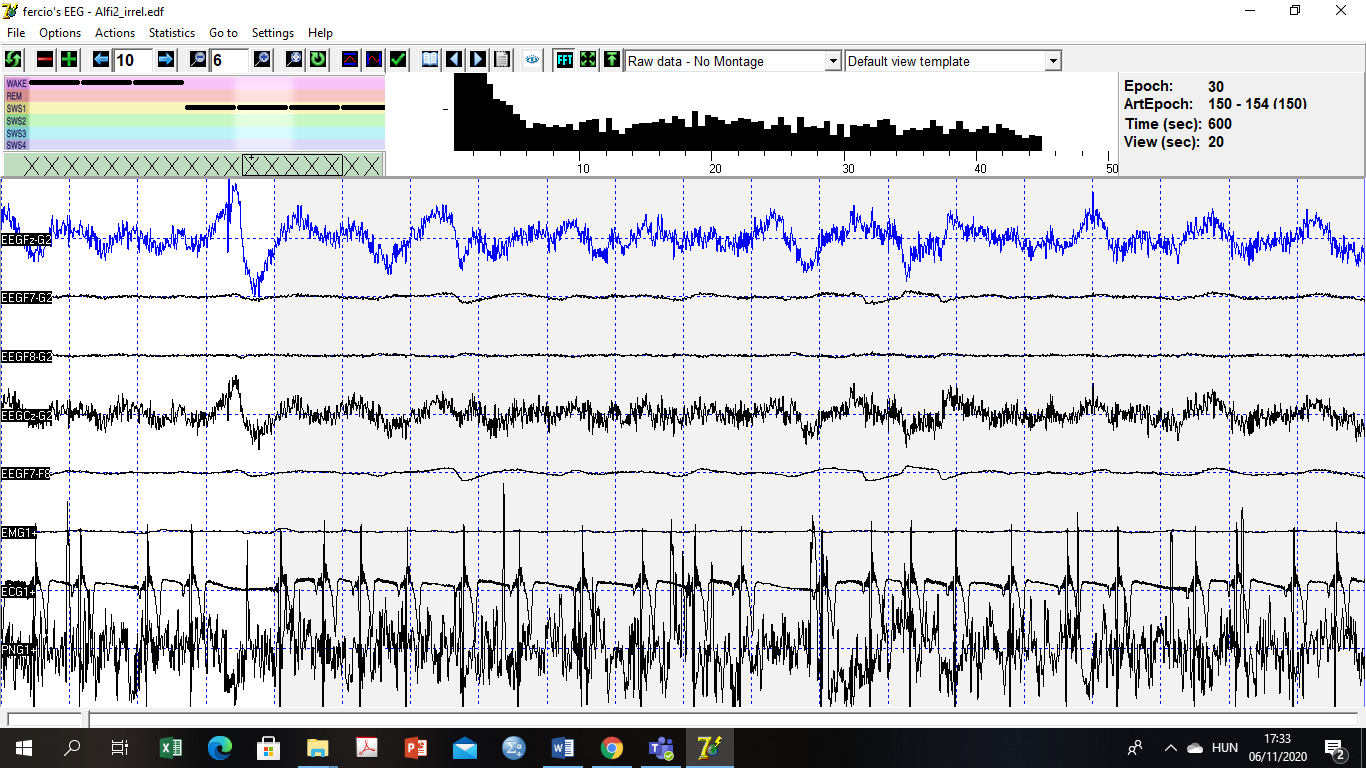


non-REM


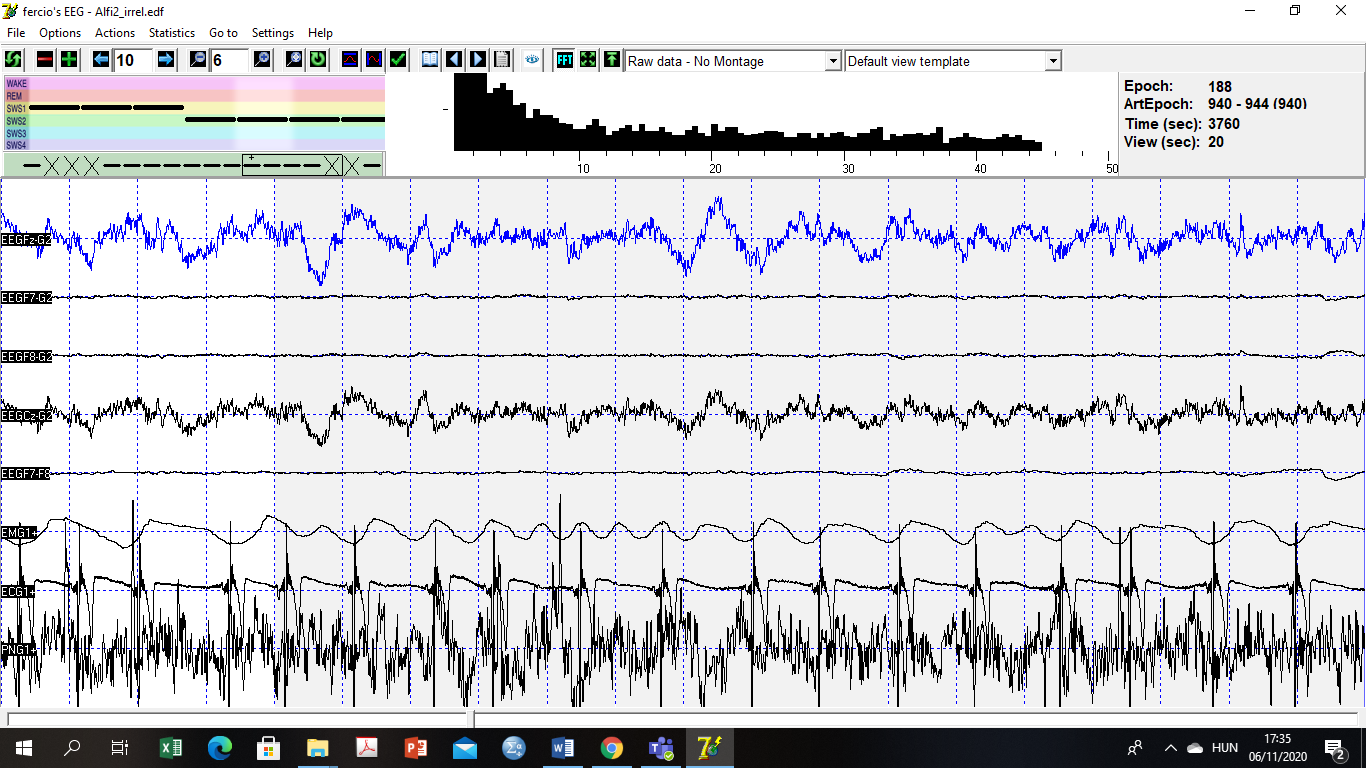


REM


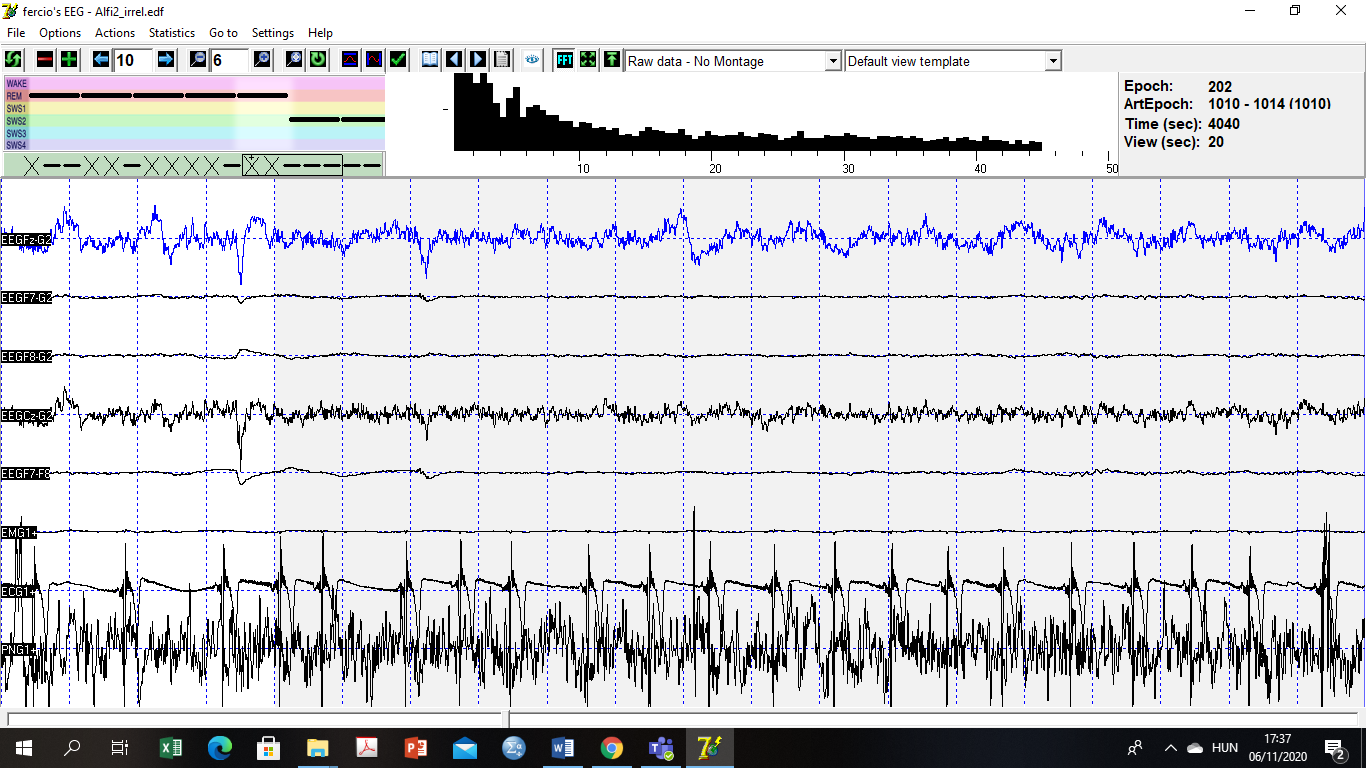


Example dog – Alfi, 6 year old, male Belgian Shepherd. Channels: Fz (EEGFz-G2), F7 (EEGF7-G2), F8 (EEGF8-G2), Cz (EEGCz-G2), Eye cannel difference (EEGF7-F8), muscle movements (EMG1+), heart (ECG1+), respiration (PNG1+).

**Non-REM sleep duration**

In data set 0 dogs spent 25.7 ± 26.4 (M ± SD) minutes in non-REM sleep during adaptation, 31.1 ± 19.2 (M ± SD, minutes non-REM) during the control condition and 41.2 ± 33.9 (M ± SD, minutes non-REM) during the learning condition.

In data set 1 dogs spent 29.4 ± 16.5 (M ± SD) minutes in non-REM sleep during adaptation, 34.8 ± 18.8 (M ± SD, minutes non-REM) during condition 1a and 44.6 ± 18.4 (M ± SD, minutes non-REM) in condition 1b.

In data set 2 dogs spent 33 ± 28.8 (M ± SD) minutes in non-REM sleep during adaptation, 47.6 ± 20.6 (M ± SD, minutes non-REM) during condition 2a and 35.4 ± 27.5 (M ± SD, minutes non-REM) during condition 2b.

**Supplementary results**

| data set/condition | electrode | r | P (two-sided) |
| --- | --- | --- | --- |
| 0 | Fz | 0.647 | 0.009 |
| 1a | Fz | 0.560 | 0.019 |
| 1a | Cz | 0.459 | 0.064 |
| 1b | Fz | -0.130 | 0.595 |
| 1b | Cz | -0.078 | 0.757 |
| 2a | Fz | 0.188 | 0.539 |
| 2a | Cz | -0.480 | 0.097 |
| 2b | Fz | -0.437 | 0.156 |
| 2b | Cz | -0.175 | 0.586 |

Table S1. Overview correlations density (spindles/minute) and learning gain (re-test - test) across data sets, conditions, and electrodes for detections in the 9-16 Hz range.

| data set/condition | electrode | spindle type | r | P (two-sided) |
| --- | --- | --- | --- | --- |
| 0 | Fz | slow | 0.688 | 0.005 |
| 0 | Fz | fast | 0.271 | 0.329 |
| 1a | Fz | slow | 0.557 | 0.020 |
| 1a | Fz | fast | 0.232 | 0.370 |
| 1a | Cz | slow | 0.458 | 0.064 |
| 1a | Cz | fast | 0.295 | 0.250 |
| 1b | Fz | slow | -0.168 | 0.491 |
| 1b | Fz | fast | 0.285 | 0.237 |
| 1b | Cz | slow | -0.165 | 0.528 |
| 1b | Cz | fast | 0.243 | 0.347 |
| 2a | Fz | slow | 0.206 | 0.501 |
| 2a | Fz | fast | -0.128 | 0.677 |
| 2a | Cz | slow | 0.149 | 0.626 |
| 2a | Cz | fast | -0.490 | 0.089 |
| 2b | Fz | slow | -0.471 | 0.122 |
| 2b | Fz | fast | 0.096 | 0.766 |
| 2b | Cz | slow | -0.660 | 0.020 |
| 2b | Cz | fast | 0.434 | 0.158 |

Table S2. Overview correlations density (spindles/minute) and learning gain (re-test - test) across data sets, conditions, and electrodes for detections classified as slow (≤ 13 Hz) or fast (≥ 13 Hz) spindles.

| data set/condition | final performance (range) | % subjects with final performance above chance (≥ 60%) | final performance (M ± SD, %) | learning gain (M ± SD, %) |
| --- | --- | --- | --- | --- |
| 0 | 61.1 - 100% | 100% | 75.2 ± 11.3 | 9.6 ± 9.7 |
| 1a | 11.1 - 83.3% | 21.1% | 48.5 ± 17.2 | 6.4 ± 22.1 |
| 1b | 0 - 77.8% | 26.3% | 41.8 ± 24.5 | -2.5 ± 16.7 |
| 2a | 33.3 - 94.4% | 38.5% | 59.8 ± 20.9 | 8.5 ± 20.5 |
| 2b | 11.1 - 94.4% | 46.2% | 56 ± 20.1 | 6.4 ± 11.3 |

Table S3. Distribution of learning performance for data sets 0, 1, and 2, including a count of how many dogs performed above chance (≥ 60%) on the final re-test (column 4).

| data set | electrode | learning measure | r | P (two-sided) |
| --- | --- | --- | --- | --- |
| 0 | Fz | learning gain | 0.575 | 0.025 |
| 0 | Fz | final performance | 0.079 | 0.780 |
| 1 | Fz | learning gain (average) | 0.152 | 0.535 |
| 1 | Fz | final performance (average) | 0.224 | 0.357 |
| 1 | Cz | learning gain (average) | 0.084 | 0.732 |
| 1 | Cz | final performance (average) | 0.202 | 0.407 |
| 2 | Fz | learning gain (average) | -0.162 | 0.596 |
| 2 | Fz | final performance (average) | 0.595 | 0.032 |
| 2 | Cz | learning gain (average) | -0.492 | 0.087 |
| 2 | Cz | final performance (average) | -0.122 | 0.691 |

Table S4. Overview of correlations between averaged density and (average) learning gain; trait density and (average) final performance, across data sets and electrodes. Averaging measures of learning success was not possible for data set 0.

| data set | electrode | learning measure | spindle type | r | P (two-sided) |
| --- | --- | --- | --- | --- | --- |
| 0 | Fz | learning gain | slow | 0.618 | 0.014 |
| 0 | Fz | learning gain | fast | 0.085 | 0.764 |
| 0 | Fz | final performance | slow | 0.197 | 0.481 |
| 0 | Fz | final performance | fast | -0.512 | 0.051 |
| 1 | Fz | learning gain (average) | slow | 0.147 | 0.549 |
| 1 | Fz | learning gain (average) | fast | 0.091 | 0.712 |
| 1 | Fz | final performance (average) | slow | 0.211 | 0.385 |
| 1 | Fz | final performance (average) | fast | 0.200 | 0.412 |
| 1 | Cz | learning gain (average) | slow | 0.075 | 0.761 |
| 1 | Cz | learning gain (average) | fast | 0.101 | 0.682 |
| 1 | Cz | final performance (average) | slow | 0.173 | 0.478 |
| 1 | Cz | final performance (average) | fast | 0.251 | 0.300 |
| 2 | Fz | learning gain (average) | slow | -0.026 | 0.934 |
| 2 | Fz | learning gain (average) | fast | -0.437 | 0.136 |
| 2 | Fz | final performance (average) | slow | 0.637 | 0.019 |
| 2 | Fz | final performance (average) | fast | -0.133 | 0.665 |
| 2 | Cz | learning gain (average) | slow | -0.373 | 0.209 |
| 2 | Cz | learning gain (average) | fast | -0.279 | 0.356 |
| 2 | Cz | final performance (average) | slow | -0.029 | 0.926 |
| 2 | Cz | final performance (average) | fast | -0.095 | 0.758 |

Table S5. Overview of correlations between averaged density and (average) learning gain; trait density and (average) final performance, across data sets and electrodes for detections classified as slow (≤ 13 Hz) and fast (≥ 13 Hz) spindles. Averaging measures of learning success was not possible for data set 0.

| sample population | spindle measure | learning measure | 95% CI | z-transformed 95% CI |
| --- | --- | --- | --- | --- |
| data set 0 | single | single | 0.2015 - 0.8707 | 0.2043 - 1.3359 |
| data set 1, condition 1a | single | single | 0.1086 - 0.8199 | 0.1090 - 1.1567 |
| data set 0 | averaged | single | 0.1144 - 0.8473 | 0.1149 - 1.2465 |
| data set 2 | averaged | averaged | 0.0655 - 0.8630 | 0.0656 - 1.3052 |

Table S6. The 95% confidence intervals for significant correlations.

**References**

1. Iotchev, I. B., Kis, A., Bódizs, R., van Luijtelaar, G. & Kubinyi, E. EEG Transients in the Sigma Range During non-REM Sleep Predict Learning in Dogs. *Sci. Rep.* **7**, 12936 (2017).

2. Iotchev, I. B. *et al.* Age-related differences and sexual dimorphism in canine sleep spindles. *Sci. Rep.* **9**, 10092 (2019).

3. Iotchev, I. B., Szabó, D., Kis, A. & Kubinyi, E. Possible association between spindle frequency and reversal-learning in aged family dogs. *Sci. Rep.* **10**, 6505 (2020).

4. Agnew, H. W., Webb, W. B. & Williams, R. L. The first night effect: An EEG study of sleep. *Psychophysiology* (1966). doi:10.1111/j.1469-8986.1966.tb02650.x

5. Reicher, V. *et al.* Repeated afternoon sleep recordings indicate first-night-effect- like adaptation process in family dogs. 1–10 (2020). doi:10.1111/jsr.12998

6. Kis, A. *et al.* Development of a non-invasive polysomnography technique for dogs (Canis familiaris). *Physiol. Behav.* **130**, 149–156 (2014).

7. Kis, A. *et al.* The interrelated effect of sleep and learning in dogs (Canis familiaris); an EEG and behavioural study. *Sci. Rep.* **7**, 41873 (2017).

8. Nonclercq, A. *et al.* Sleep spindle detection through amplitude-frequency normal modelling. *J. Neurosci. Methods* **214**, 192–203 (2013).
